# Dynamic RNA Polymerase compartments organize the transcription of gene clusters

**DOI:** 10.64898/2026.01.07.698080

**Published:** 2026-01-08

**Authors:** Yi-Hui Wang, Hannah L. Hertz, Wen Tang

**Affiliations:** 1 Department of Biological Chemistry and Pharmacology, The Ohio State University, Columbus, OH 43210, USA; 2 Center for RNA Biology, The Ohio State University, Columbus, OH 43210, USA; 3 Ohio State Biochemistry Program, The Ohio State University, Columbus, OH 43210, USA

## Abstract

Spatial organization of transcription machinery is emerging as a key regulator of gene expression, yet how RNA polymerases are organized at gene clusters remains unclear. Here, we show that RNA polymerases II and polymerase III form distinct nuclear foci at the 5S ribosomal DNA (rDNA)–Spliced leader 1 (SL1) cluster in *C. elegans*. Within this cluster, polymerase II binds to the SL1 gene, while polymerase III associates with 5S rDNA. Both polymerase foci display dynamic but distinct behaviors within the nucleus. The assembly of these polymerase foci is regulated across the cell cycle. ATTF-6, an AT-hook transcription factor, is essential for polymerases II foci formation but dispensable for polymerases III foci. While Pol III foci are largely resistant to temperature changes, Pol II foci are temperature-sensitive, and their dissolution correlates with reduced SL1 expression. Together, these results reveal a spatial and temporal regulation of two RNA polymerases that organize gene cluster transcription.

## Introduction

In eukaryotes, transcription is carried out by three major RNA polymerases, each dedicated to distinct classes of genes. RNA polymerase I (Pol I) transcribes ribosomal RNAs (rRNAs), polymerase II (Pol II) produces messenger RNAs (mRNAs) and many long non-coding RNAs, and RNA polymerase III (Pol III) synthesizes transfer RNAs (tRNAs), 5S rRNA, and other small structural RNAs (Cramer 2019; Roeder 2019; Girbig et al. 2022). Beyond diffuse nucleoplasmic distributions, accumulating evidence indicates that transcription is spatially organized into focal compartments or condensates that coordinate efficiency and specificity (Rippe 2022; Rippe and Papantonis 2025).

RNA Pol I is highly enriched in the nucleolus, a membraneless compartment specialized for rRNA biogenesis (Grummt 2003). In contrast, Pol II is expressed throughout the nucleoplasm. Pol II transcription can occur within discrete “transcription factories” or nuclear foci (Jackson et al. 1993; Wansink et al. 1993). These foci represent sites where multiple genes, transcription factors, and nascent transcripts are locally concentrated, providing a framework for coordinated transcription and RNA processing. Super-resolution imaging has shown that a substantial fraction of Pol II resides in these foci, and emerging models suggest that liquid–liquid phase separation and related microphase separation processes underlie the formation and dynamics of transcriptional condensates (Banani et al. 2017; Hnisz et al. 2017; Rippe 2022). Proteins involved in transcription, such as Mediator components, transcription factors, and the intrinsically disordered C-terminal domain of Pol II, can form multivalent weak interactions that promote condensate assembly (Hnisz et al. 2017; Boehning et al. 2018; Cho et al. 2018; Palacio and Taatjes 2022). However, the mechanisms governing transcriptional condensate formation and their biological relevance remain actively debated (McSwiggen et al. 2019; Stortz et al. 2024; Rippe and Papantonis 2025).

Compared to Pol I and Pol II, the spatial organization of Pol III is less understood. Pol III transcribes non-coding RNAs such as tRNAs and 5S rRNAs, both of which are essential for translation. Studies suggest that its activity may concentrate at specific nuclear foci, analogous to Pol II (Pombo et al. 1999). Yet, the spatial relationship between Pol III, Pol II and other transcriptional machineries, and how these polymerase-specific compartments are regulated, remain largely unknown.

Many organisms organize functionally related genes into clusters, including piRNA, histone, and rRNA genomic loci (Blumenthal 1998; Duronio and Marzluff 2017; Pastore et al. 2022). In *C. elegans*, the 5S rDNA–spliced leader 1 (SL1) cluster offers a unique opportunity to study how Pol II and Pol III transcriptional programs coexist in close proximity. This cluster consists of more than 100 tandem ~1 kb repeats on chromosome V, each unit harboring one 5S rDNA gene and one SL1 gene (Ellis et al. 1986; Ding et al. 2022). The 5S rRNA is transcribed by Pol III whose largest subunit is RPC-1 in *C. elegans*, while SL1 is transcribed by Pol II with AMA-1 as its catalytic subunit (Rogalski and Riddle 1988; Bird and Riddle 1989). SL1 encodes a specialized snRNA (small nuclear RNA) required for trans-splicing, a process that appends SL1 to the 5′ ends of more than half of all pre-mRNAs to promote efficient translation initiation (Blumenthal 2012; Yang et al. 2017). Despite its biological importance, the transcriptional regulation and nuclear organization of the 5S rDNA–SL1 cluster remain poorly characterized.

Here, we exploit this dual-polymerase gene cluster to investigate how two distinct RNA polymerases form spatially organized transcription compartments in vivo. We show that Pol II and Pol III form separate, dynamic nuclear foci at the 5S rDNA–SL1 cluster, each regulated in a cell cycle–dependent manner. Pol II foci require the transcription factor ATTF-6, are sensitive to temperature, and regulate SL1 expression, whereas Pol III foci are less affected by these conditions. Together, our findings reveal that *C. elegans* RNA polymerases assemble into distinct, condensate-like compartments at shared gene clusters, uncovering an unexpected layer of spatial organization that coordinates transcription across polymerase systems.

## Results

### RNA Pol II and Pol III form distinct nuclear foci at the 5S rDNA–SL1 cluster

Our recent work showed that ATTF-6, an AT-hook transcription factor, associates with genomic clusters including the piRNA cluster and the 5S rDNA–SL1 cluster in the *C. elegans* gonad (Wang et al. 2025). The gonad is a syncytial tissue in which germ cells proliferate mitotically at the distal end and then enter meiotic pachytene to produce oocytes, which are fertilized to form embryos (Figure S1A) (Pazdernik and Schedl 2013). ATTF-6 forms a large nuclear focus at the piRNA cluster specifically in the early–mid pachytene region, where homologous chromosomes undergo synapsis (Figures S1B and S1C) (Batista et al. 2008; Das et al. 2008; Wang et al. 2025). Furthermore, ATTF-6 forms a smaller and discrete focus at the 5S rDNA–SL1 cluster throughout pachytene and oogenesis (Figures S1B and S1C) (Wang et al. 2025).

**Figure 1.**
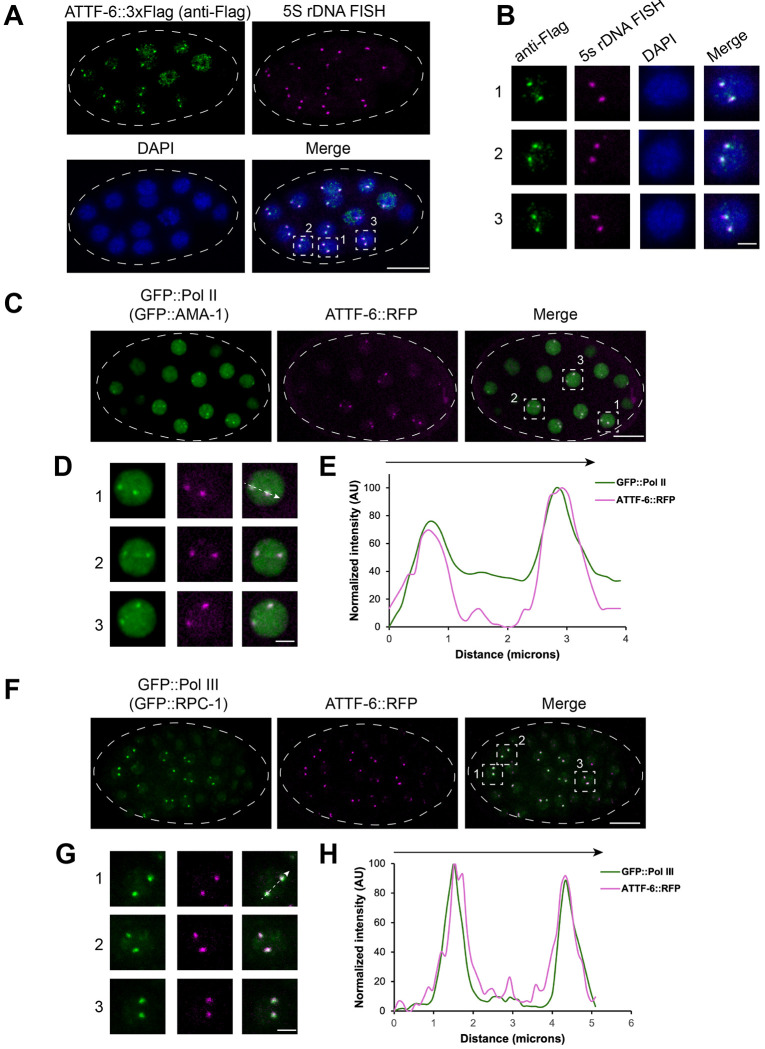
RNA Pol II and Pol III form distinct nuclear foci at the 5S rDNA-SL1 gene cluster. **(A)** Co-localization of 5S rDNA and ATTF-6 in embryo nuclei detected by DNA-FISH and immunostaining. DNA-FISH was performed using Cy5-labeled probes targeting the 5S rDNA gene cluster, and immunostaining was conducted with an anti-FLAG antibody to detect ATTF-6::3×FLAG. Nuclei were visualized with DAPI. Dashed lines outline the embryo. Confocal images (60× objective) are shown as maximum-intensity projections spanning the top layer of nuclei in the fixed embryo. Scale bar: 10 μm. **(B)** Enlarged view of three nuclei within the embryo shown in (**A**). Scale bar: 2 μm. **(C)** GFP::Pol II (GFP:: AMA-1) and ATTF-6::RFP foci in a live embryo. Dashed lines outline the embryo. Confocal images (60× objective) are shown as maximum-intensity projections spanning the top layer of nuclei in the embryo. Scale bar: 10 μm. **(D)** Enlarged view of three nuclei within the embryo shown in (**C**). Scale bar: 2 μm. **(E)** Intensity profile of GFP::Pol II (GFP::AMA-1) and ATTF-6::RFP signals along the dotted arrow in nucleus No1. in (**D**). AU, arbitrary unit. **(F)** GFP::Pol III (GFP::RPC-1) and ATTF-6::RFP foci in a live embryo. Dashed lines outline the embryo. Confocal images (60× objective) are shown as maximum-intensity projections spanning the top layer of nuclei in the embryo. Scale bar: 10 μm. **(G)** Enlarged view of three nuclei within the embryo shown in (**F**). Scale bar: 2 μm. **(H)** Intensity profile of GFP::Pol III (GFP::RPC-1) and ATTF-6::RFP signals along the dotted arrow in nucleus No. 1 in (**G**). AU, arbitrary units.

To extend these observations to early embryos, we examined the localization of endogenously tagged ATTF-6. We performed 5S rDNA FISH (Fluorescence in situ hybridization) followed by immunofluorescence against ATTF-6::3xFLAG using an anti-FLAG antibody (Figures 1A and 1B). ATTF-6::3xFLAG formed discrete nuclear foci in embryos that colocalized with the 5S rDNA–SL1 cluster indicated by 5S rDNA FISH signals (Figures 1A and 1B). These results indicate that ATTF-6 associates with the cluster not only in germ cells but also in embryonic nuclei.

The 5S rDNA–SL1 cluster is unique in that it contains over 100 tandem copies of the 5S rRNA gene and SL1 snRNA gene (Ellis et al. 1986; Ding et al. 2022). The 5S rRNA, transcribed by Pol III, is an essential component of ribosomes, while SL1, transcribed by Pol II, provides the spliced leader sequence for trans-splicing (Blumenthal 2012; Yang et al. 2017). The presence of two polymerase-dependent transcription units within a highly repetitive genomic region raises the question of whether Pol II and Pol III are locally enriched at this cluster.

To address this, we examined the subcellular localization of GFP::AMA-1 (the largest catalytic subunit of Pol II) and GFP::RPC-1 (the largest catalytic subunit of Pol III) expressed from their endogenous genomic loci. Both polymerases were broadly distributed throughout nuclei, but showed modest and noticeable enrichment at the small ATTF-6::RFP foci corresponding to the 5S rDNA–SL1 cluster in the gonad (Figures S1B and S1C). Neither polymerase was enriched at piRNA clusters marked by mCherry::PRDE-1, a piRNA specific transcription factor (Figures S1D and S1E) (Weick et al. 2014; Weng et al. 2019; Wang et al. 2025).

In embryos, confocal microscopy revealed that GFP::Pol II formed several nuclear foci, including prominent two that colocalized with ATTF-6::RFP (Figures 1C and 1D), as confirmed by line-scan intensity profiles (Figure 1E). Similarly, GFP::Pol III formed two prominent foci in embryos that overlapped with ATTF-6 (Figures 1F–1H). These data demonstrate that Pol II and Pol III assemble into distinct nuclear foci at the 5S rDNA–SL1 cluster in both the germline and early embryos.

### Pol II associates with SL1 gene while Pol III associates with 5S rDNA

**Figure 2.**
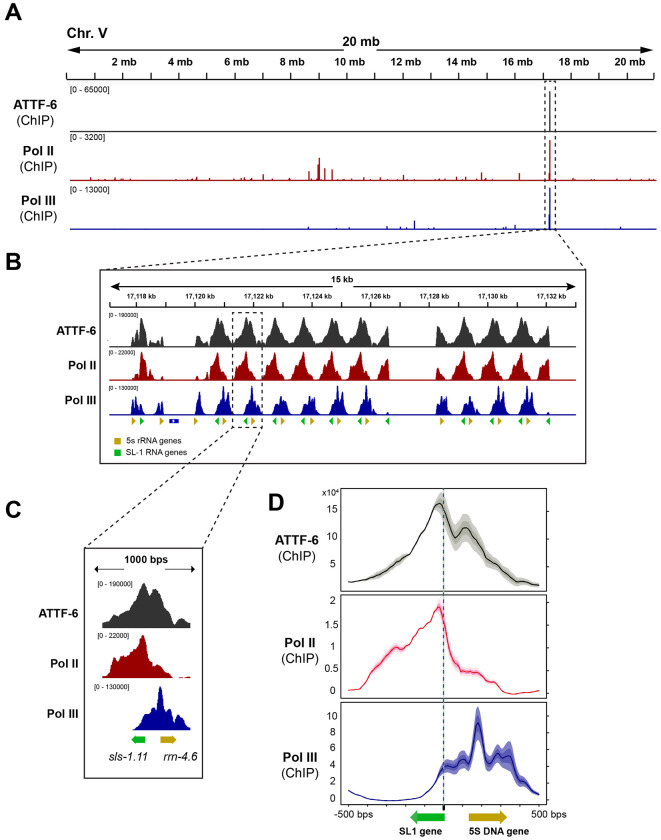
RNA Pol II and Pol III associate with SL1 and 5S rDNA, respectively. **(A)** Browser view of ATTF-6, Pol II (AMA-1), and Pol III (RPC-1) ChIP-seq signals on chromosome V. ATTF-6 ChIP-seq signals were normalized to the control IP (Wang et al. 2025)., and AMA-1 and RPC-1 ChIP-signals were normalized to the input (Ikegami and Lieb 2013). The signals represent the average from two biological replicates and are shown in RPKM (Reads Per Kilobase per Million mapped reads). The dashed box highlights the 5S rDNA–SL-1 gene cluster. **(B)** Browser view zoomed in on the 5S rDNA–SL1 gene cluster. The cluster contains more than 100 tandem repeats of 5S rDNA (yellow arrowhead) and SL1 (green arrowhead) arranged in opposite orientations. **(C)** An individual example within the 5S rDNA–SL1 gene cluster. The green and yellow arrows indicate the positions and orientations of *sls-1.11* and *rrn-4.6*, respectively. **(D)** Metagene profiles of ATTF-6, Pol II (AMA-1), and Pol III (RPC-1) ChIP-seq signals around the SL1 snRNA and 5s rRNA genes. The plot is anchored at the 5’ end of the SL1 snRNA genes and spans 500 bp upstream and downstream. Signals (RPKM) were normalized to the control by subtraction and then averaged across two biological replicates. Yellow and green arrows indicate the positions and orientations of the 5S rRNA and SL1 snRNA genes, respectively.

To further validate the enrichment of polymerases at the 5S rDNA–SL1 cluster, we reanalyzed published ChIP-seq (Chromatin Immunoprecipitation followed by high-throughput sequencing) datasets (Ikegami and Lieb 2013). Specifically, we examined ATTF-6, Pol II and Pol III ChIP signals across individual chromosomes including five autosomes and one sex chromosome (chromosome X). Consistent with our previous findings (Wang et al. 2025), genome-wide profiles showed that ATTF-6 binds strongly to the piRNA cluster on Chromosome IV and to the 5S rDNA–SL1 cluster on Chromosome V (Figures 2A and S2). Importantly, the single strongest Pol II and Pol III ChIP peaks across the genome occurred at the 5S rDNA–SL1 locus (Figures 2A, 2B and S2).

A browser view of a single repeat with *sls-1.11* and *rrn-4.6* showed that ATTF-6 binds to the promoter region shared by the 5S rDNA and SL1 genes, with peak enrichment nearer to the SL gene (Figure 1C). Pol II was sharply enriched at *sls-1.11* (SL1 gene), while Pol III was strongly enriched at *rrn-4.6* (5S rDNA gene) (Figure 1C). Metagene analysis centered on the SL1 TSS (Transcription Start Site) further supported such organization: ATTF-6 peaked near TSS of SL1, Pol II was highest directly over SL1, and Pol III peaked over the 5S rDNA gene body (Figure 1D). Collectively, ChIP-seq and fluorescence microscopy data demonstrate that Pol II and Pol III each form distinct nuclear foci that correspond to their respective transcriptional targets at the 5S rDNA–SL1 cluster.

### Pol II and Pol III foci are dynamic and exhibit properties of liquid-like condensates

Previous studies suggest that Pol II and several transcription factors form liquid-like condensates, potentially through liquid–liquid phase separation (Hnisz et al. 2017; Boehning et al. 2018; Cho et al. 2018; Palacio and Taatjes 2022). We therefore asked whether Pol II and Pol III foci at the 5S rDNA–SL1 cluster display hallmark features of biomolecular condensates. At least three commonly used criteria include: (1) a 2-dimensional rounded or 3-dimensional spherical morphology driven by the surface tension, (2) Dynamic molecular exchange with the surrounding environment, and (3) Although variably observed, sensitivity to aliphatic alcohols such as 1,6-hexanediol which disrupt weak hydrophobic interactions (Brangwynne et al. 2009; Patel et al. 2015; Alberti et al. 2019).

**Figure 3.**
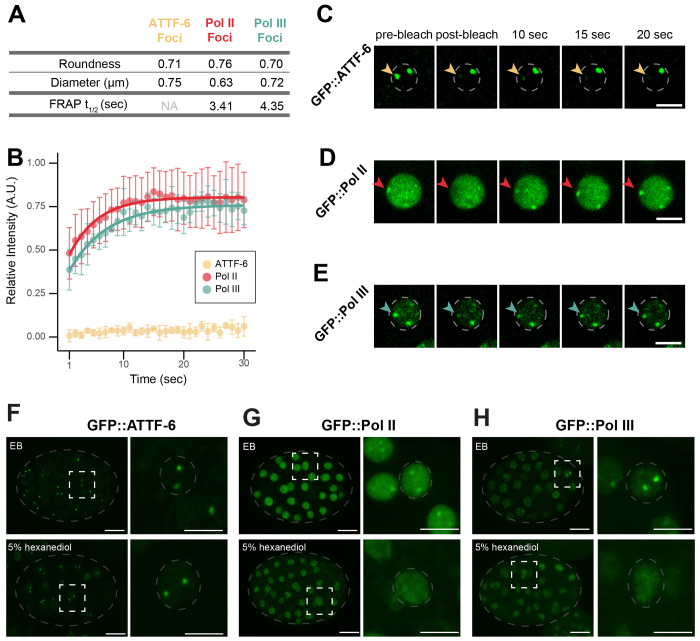
RNA Pol II and Pol III form dynamic foci *in vivo*. **(A)** Table showing foci shape descriptors (top rows), calculated from maximum-intensity projections (60x) for three GFP::ATTF-6, GFP::Pol II, or GFP::Pol III embryos and fluorescence recovery after photobleaching (FRAP) parameters—t_1/2_ (bottom rows), calculated from models shown in (**B**) for GFP::ATTF-6, GFP::Pol II, and GFP::Pol III. FRAP parameters for ATTF-6 are labeled as NA (not applicable) because the recovery did not fit the exponential model. **(B)** Relative intensity of GFP::ATTF-6 (n=4), GFP::Pol II (n=6) and GFP::Pol III (n=4) foci, respectively, after photobleaching in embryos. Intensity was measured from single-plane images every second (sec) before and after photobleaching and normalized to the mean intensity over five timepoints prior to bleaching (time 0). A.U. indicates arbitrary units. Dots represent mean relative intensity, and error bars denote standard deviation. Red and blue curves represent the exponential model of recovery for Pol II or Pol III, respectively. **(C)** Single-plane time-lapse (100x) images of GFP::ATTF-6 before (pre-bleach) and after photobleaching for the indicated timepoints (seconds). Yellow arrow indicates the photobleached focus; dashed circle indicates the nuclear periphery. Scale bar: 5 μm. **(D)** Same as in (**C**) but for GFP::Pol II. Red arrow indicates the photobleached focus. Scale bar: 5 μm. **(E)** Same as in (**C**) but for GFP::Pol III. Blue arrow indicates the photobleached focus; dashed circle indicates the nuclear periphery. Scale bar: 5 μm **(F-H)** Representative maximum intensity projections (60x) of GFP::ATTF-6 (**F**), GFP::Pol II (**G**), or GFP::Pol III (**H**) expressing embryos treated with *ptr-2* RNAi and subsequently dissected into either egg buffer (EB) or 5% 1,6-hexanediol. Images depict whole embryos, with each embryo outlined by grey dashed lines (left; scale bar: 10 μm) and the corresponding inset indicated by the white dashed square (right; inset scale bar: 5 μm). Grey dashed circles in each inset outline the nucleus.

We first quantified the morphology of ATTF-6, Pol II, and Pol III foci in embryos using three-dimensional reconstructions. Roundness values (with 1 representing a perfect sphere) were 0.71 for GFP::ATTF-6 foci and 0.70 for GFP::Pol II foci, whereas GFP::Pol III foci were slightly more spherical, with a mean value of 0.76 (Figure 3A). The mean diameter was ~630 nm for Pol II foci and ~720 nm for Pol III foci, both of which exceeded the diffraction limit of our fluorescence microscopy (Figure 3A).

We next assessed molecular dynamics using FRAP (Fluorescence Recovery After Photobleaching). In brief, ATTF-6, Pol II, or Pol III foci were photobleached and fluorescence recovery was monitored over 30 seconds. Recovery curves for Pol II and Pol III were well described by a single-exponential model, while ATTF-6 recovery could not be fit by this model (Figures 3A–3E). We found that Pol II and Pol III foci displayed much faster and more complete recovery than ATTF-6 foci (Figures 3A–3E). Specifically, the half-time of recovery (t1/2) was 3.41 seconds for Pol II, and 4.35 seconds for Pol III (Figure 3A). Thus, Pol II and Pol III foci exhibit rapid molecular exchange with the surrounding nucleoplasm when compared to ATTF-6.

Finally, because 1,6-hexanediol disrupts certain liquid-like condensates by interfering with weak hydrophobic interactions (Alberti et al. 2019; Zheng et al. 2025), we tested the effect of 1,6-hexanediol on ATTF-6, Pol II and Pol III foci. Hexanediol treatment did not dissolve ATTF-6 foci (Figure 3F). In contrast, Both Pol II and Pol III foci were disrupted following treatment (Figures 3G and 3H). Overall, these data indicate that ATTF-6 foci are relatively static, whereas Pol II and Pol III foci display spherical morphology and dynamic exchange consistent with features of biomolecular condensates.

### Formation of Pol II and Pol III foci is regulated across the cell cycle

**Figure 4:**
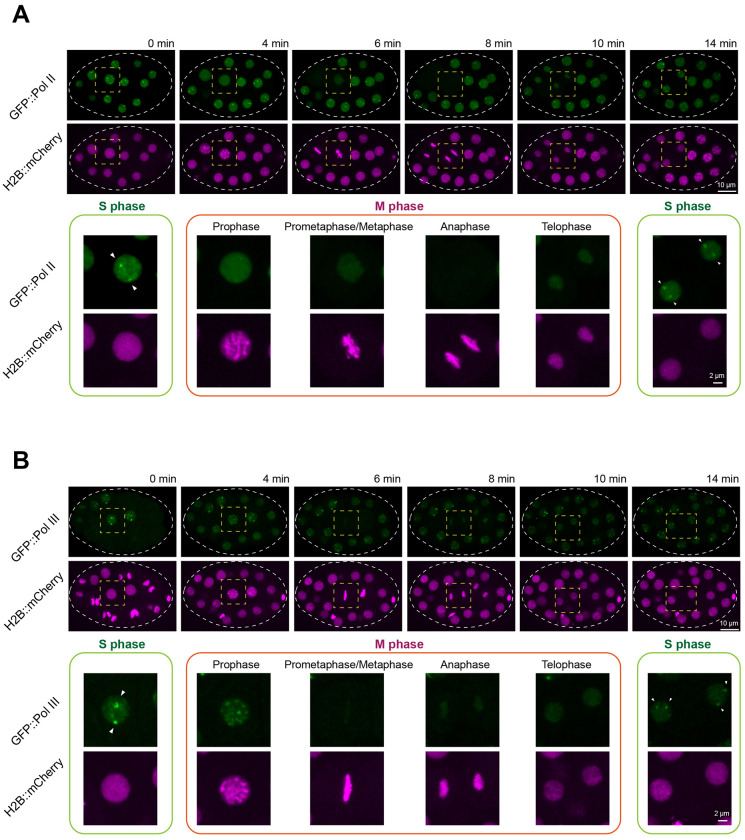
Formation of Pol II and Pol III foci is regulated across the cell cycle. **(A)** Dynamics of GFP::Pol II foci during cell division in an early embryo. A time-lapse imaging series of an embryo expressing GFP::Pol II and the chromatin marker H2B::mCherry. The circular dashed line outlines the embryo, and the dashed square highlights the dividing cell. The bottom panel shows zoomed-in views of the dividing cell, grouped into S phase and M phase. Arrowheads indicate GFP::Pol II foci in the nucleus of the dividing cell. Confocal images (60× objective) are shown as maximum-intensity projections spanning the top layer of nuclei in the embryo. Scale bars: 10 μm (top panel) and 2 μm (bottom panel). **(B)** Same analysis as in (**A**), but with GFP::Pol III.

We noticed that not all embryonic cells exhibited Pol II or Pol III foci (Figures 1 and 3), indicating that foci formation might depend on the cell cycle. Early *C. elegans* embryonic divisions consist only of S phase and M phase (Kipreos and van den Heuvel 2019). To monitor these cell-cycle stages, we used fluorescently tagged histone (H2B::mCherry), which appears diffuse within the nucleus during S phase and becomes bright and highly condensed as chromosomes entering mitosis (Figures 4A and 4B).

Using this H2B::mCherry marker, we followed the dynamics of Pol II and Pol III foci within a single embryonic cell cycle (~14 minutes). Pol II foci were consistently observed during S phase (Figure 4A). As the cell entered prophase, Pol II foci diminished in intensity and fully dispersed during mitosis. After division, Pol II foci reappeared rapidly in the S phase of each daughter cell (Figure 4A).

Pol III foci displayed a similar dependence on cell-cycle stage but with slightly distinct mitotic behavior. Pol III foci were present during S phase (Figure 4B). Upon entry into mitosis, Pol III signals redistributed onto condensed chromosomes and then became diffuse from metaphase through telophase. As with Pol II, Pol III foci re-formed in the next S phase (Figure 4B). Together, these observations demonstrate that Pol II and Pol III foci are tightly regulated by the cell cycle: both assemble during S phase, disperse during mitosis, and reassemble in the following interphase.

### ATTF-6 is required for Pol II foci formation but is dispensable for Pol III foci

**Figure 5.**
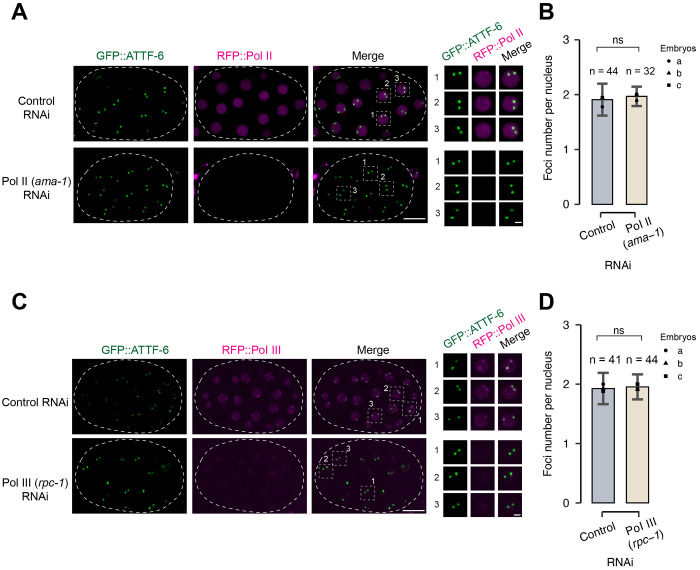
Pol II nor Pol III are dispensable for assembly of ATTF-6 foci. **(A)** Fluorescence micrographs of nuclei in live embryos expressing GFP::ATTF-6 and RFP::Pol II. Embryos were dissected from worms treated with control (L4440) or Pol II (*ama-1*) RNAi. The circular dashed line outlines the embryo. Images are shown as maximum-intensity projections spanning the top nuclear layer. The right panel presents individual enlarged views of three nuclei selected from the embryo. Confocal images were acquired with a 60× objective. Scale bars: 10 μm (embryo) and 2 μm (nuclei). (**B**) Quantification of GFP::ATTF-6 foci numbers in embryo nuclei. Embryos were dissected from worms treated with control (L4440) or Pol II (*ama-1*) RNAi. Nuclei from three independent embryos were counted. Error bars represent the standard deviation (SD). Statistical significance was determined using a two-tailed Student’s t-test (ns: p > 0.05). **(C-D)** Analyses as in (**A-B**), but using embryos dissected from worms treated with Pol III (*rpc-1*) RNAi.

Because ATTF-6 colocalizes with both Pol II and Pol III at the 5S rDNA–SL1 cluster (Figure 1), we asked whether the formation of ATTF-6 foci and polymerase foci are interdependent. To test whether polymerase activity acts upstream of ATTF-6, we examined ATTF-6 localization following depletion of Pol II or Pol III using RNAi (RNA interference) (Fire et al. 1998; Kamath et al. 2003). In embryos expressing GFP::ATTF-6 and RFP::Pol II from their endogenous loci, RNAi against Pol II efficiently reduced RFP::Pol II signals (Figures 5A and 5B). However, it had no effect on GFP::ATTF-6 foci (Figures 5A and 5B). Likewise, depletion of Pol III did not alter ATTF-6 foci formation (Figures 5C and 5D). These results indicate that neither Pol II nor Pol III is required for assembly of ATTF-6 foci.

**Figure 6.**
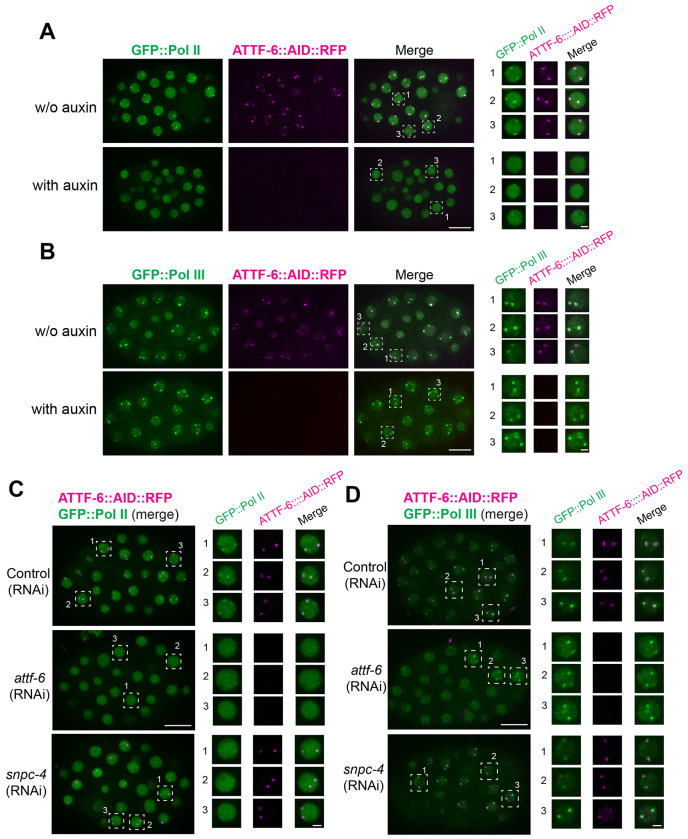
SNPC-4 and ATTF-6 are required for Pol II foci, but not Pol III foci formation. **(A)** Live embryos expressing GFP::Pol II and ATTF-6::AID::RFP, dissected from worms treated with or without 1 mM auxin. The right panel presents individual enlarged views of three nuclei selected from each embryo. Confocal images (60× objective) are shown as maximum-intensity projections spanning the top nuclear layer. Scale bars: 10 μm (embryo) and 2 μm (nuclei). **(B)** Same auxin treatment conditions as in **(A)**, but using embryos expressing GFP::Pol III and ATTF-6::AID::RFP. **(C)** Live embryos expressing GFP::Pol II and ATTF-6::AID::RFP, dissected from worms treated with control (L4440), *snpc-4*, or *attf-6* RNAi. Embryos show the merged GFP and RFP signals. The right panels present individual enlarged views of three nuclei selected from each embryo, shown as separate GFP, RFP, and merged channels. Confocal images (60× objective) are shown as maximum-intensity projections spanning the top nuclear layer. Scale bars: 10 μm (embryo) and 2 μm (nuclei). **(D)** Same RNAi conditions as in (**C**), but showing embryos expressing GFP::Pol III together with ATTF-6::AID::RFP.

We next performed the reciprocal test to determine whether ATTF-6 is required for polymerase foci formation. Since *attf-6* is an essential gene (Wang et al. 2025), we depleted ATTF-6 protein using the auxin-inducible degron (AID) system (Zhang et al. 2015). Using CRISPR/Cas9 gene editing, we tagged ATTF-6 at its endogenous locus with RFP and AID, enabling both visualization and auxin-dependent degradation. In the absence of auxin, GFP::Pol II formed two prominent nuclear foci that colocalized with ATTF-6::AID::RFP (Figures 6A). Upon auxin treatment, ATTF-6::AID::RFP foci became undetectable (Figures 6A, S3A and S3B). Depletion of ATTF-6 resulted in the loss of large Pol II foci (Figures 6A, S3A and S3B). In contrast, depletion of ATTF-6 had no robust effect on Pol III foci, when compared to the untreated control (Figures 6B, S3A and S3C). These findings demonstrate that ATTF-6 is required for Pol II, but not Pol III, foci formation.

To further test this requirement and to identify additional factors involved in polymerase foci assembly, we used RNAi to deplete *attf-6* as well as *snpc-4*, a subunit of Small Nuclear RNA-activating Protein Complex that drives the transcription of snRNA genes (Kasper et al. 2014; Hou et al. 2022). Consistent with AID-mediated depletion, RNAi knockdown of *attf-6* caused dispersal of Pol II foci without affecting Pol III foci (Figures 6C, 6D, S3D, S3E). Knockdown of *snpc-4* did not disrupt ATTF-6 localization. However, it selectively impaired Pol II foci formation, while Pol III foci remained intact (Figures 6C, 6D, S3D, S3E). Together, these results show that ATTF-6 and SNPC-4 are specifically required for the formation of Pol II foci at the 5S rDNA–SL1 cluster, while Pol III foci assemble independently of these factors.

### Pol II foci are temperature-sensitive and regulate SL1 expression

To investigate the functional significance of polymerase foci in regulating SL1 and 5S rRNA expression, we examined how their assembly responds to environmental cues. *C. elegans* is a poikilothermic organism whose cellular processes adapt to environmental temperature. And biomolecular condensates are often sensitive to thermal perturbation (Fritsch et al. 2021; Alberti et al. 2025). We therefore tested whether elevated temperature affects the formation of ATTF-6, Pol II, or Pol III foci.

**Figure 7.**
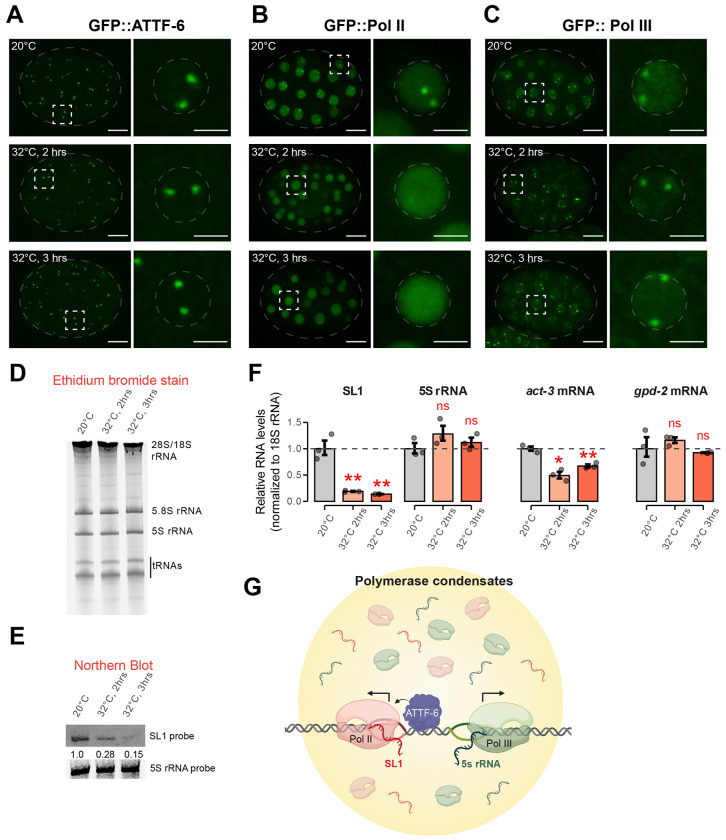
Pol II foci are temperature-sensitive and regulate SL1 expression. **(A)** Representative maximum intensity projections (60x) of GFP::ATTF-6 expressing embryos cultured at 20°C (top) or shifted to 32°C for either 2 hours (middle) or 3 hours (bottom). Whole embryos are shown on the left (scale bar: 10 μm), outlined by grey dashed ovals. The corresponding insets to the right are indicated by the white dashed squares. Insets to the right show single nuclei outlined by grey dashed circles (Inset scale bar: 3 μm). Heating conditions are further described in the Materials and Methods. **(B)** Same conditions as in (**A**), but for GFP::Pol II expressing embryos. **(C)** Same conditions as in (**A**), but for GFP::Pol III expressing embryos **(D)** Ethidium bromide staining of total RNAs from wild-type embryos cultured at 20°C, or shifted to 32°C for either 2 hours or 3 hours in M9 buffer, respectively. rRNAs and tRNAs are indicated. **(E)** Northern blotting of SL1 RNA (top) or 5S rRNA (bottom) from wild-type embryos cultured at 20°C, or shifted to 32°C for either 2 hours or 3 hours, respectively. Relative SL1 abundance upon heating was normalized to 5S rRNA abundance. **(F)** RT-qPCR analysis of Pol II transcripts (SL1, *act-3*, and *gpd-2)* and the Pol III transcript 5S rRNA in embryos. Transcript fold change upon heat stress was calculated relative to 20°C and normalized to 18S rRNA as the internal control. Bar plots show mean +/− SD; dots represent technical replicates. Statistical significance was assessed using Student’s two-sample t-test. * indicates p ≤0.05, ** p ≤0.01, and ns indicates not significant. **(G)** Working model showing Pol II and Pol III occupy the 5S rDNA–SL1 locus but assemble into distinct nuclear compartments. ATTF-6 promotes Pol II foci formation to drive SL1 transcription while Pol III independently engages nearby 5S rRNA genes.

Embryos were shifted from 20 °C to 32 °C, and imaged after 2 and 3 hours. GFP::ATTF-6 foci remained intact under these mild heat stress conditions (Figures 7A and S4A). In contrast, Pol II foci were highly temperature-sensitive: GFP::Pol II foci dissolved at 32 °C, and most embryonic nuclei completely lost Pol II foci within 3 hours (Figures 7B and S4B). GFP::Pol III foci, however, remained intact at 32 °C (Figures 7C and S4C). These observations demonstrate that only Pol II foci exhibit temperature-dependent disassembly, while ATTF-6 and Pol III foci are temperature-insensitive.

We next investigated whether temperature-dependent changes in polymerase foci correlate with altered SL1 or 5S rRNA expression. Total RNA extracted from embryos incubated at 20 °C or 32 °C was analyzed by denaturing gel electrophoresis. Ethidium bromide staining revealed no detectable change in the abundance of major RNA species including tRNAs, 5S, 5.8S, 18S, and 28S rRNAs (Figure 7D). Northern blotting of the same samples demonstrated that SL1 RNA levels decreased ~4-fold after 2 h at 32 °C and ~5-fold after 3 h, when normalized to 5S rRNA signals (Figure 7E). RT–qPCR analysis corroborated these results: SL1 transcripts were significantly reduced under the heat stress, while 5S rRNA levels were unaffected when normalized to 18S rRNA (Figure 7F). Among housekeeping controls, *act-3* mRNA showed a modest decrease, while *gpd-2* levels were unchanged (Figure 7F). Together, these findings indicate that elevated temperature selectively disrupts Pol II foci and that this disassembly correlates with a marked reduction in SL1 transcription.

## Discussion

Although the spatial organization of transcription machinery is increasingly recognized as a key regulatory layer, a major knowledge gap remains: it is unclear whether different RNA polymerases form distinct compartments when operating within the same gene cluster, and how such organization influences transcriptional output. In this study, we uncover a specialized nuclear microenvironment at the *C. elegans* 5S rDNA–SL1 cluster in which RNA polymerases II and III form distinct and dynamic compartment. Our findings support a model in which transcription factors including ATTF-6 and SNPC-4 recruit or stabilize Pol II at the SL1 gene cluster to promote efficient SL1 transcription, while Pol III independently assembles on adjacent 5S rRNA genes (Figure 7G). These two polymerases therefore occupy the same genomic locus but form biochemically and functionally distinct nuclear bodies.

An emerging theme in gene regulation is that transcription can be organized into spatially restricted structures, ranging from transient hubs of polymerase-transcription factor interactions to fully formed biomolecular condensates generated by phase separation (Banani et al. 2017; Hnisz et al. 2017; Rippe 2022). Our data place Pol II and Pol III foci at the 5S rDNA–SL1 cluster closer to the latter category. Both foci are approximately spherical and display rapid fluorescence recovery after photobleaching. Furthermore, Pol II foci are sensitive to 1,6-hexanediol and thermal perturbation. These features match the operational hallmarks of liquid-like condensates. However, these polymerase foci differ from previously reported Pol II condensates (Cho et al. 2018). They are unusually large (> 600 nm), highly localized to a specific gene cluster, and assemble in a cell-cycle–dependent manner strictly during S phase. This suggests that the polymerase bodies at the 5S rDNA–SL1 locus represent a specialized class of “gene-specific compartment” tuned to the unique regulatory needs of these small non-coding RNA genes.

Although Pol II and Pol III foci occupy the same genomic region, their formation is mechanistically separable. Pol III foci assemble independently of ATTF-6 and SNPC-4 and are markedly more stable under heat stress. Pol II foci, in contrast, require ATTF-6 and SNPC-4 and are the only polymerase foci sensitive to elevated temperature. These differences reveal distinct regulatory architectures for the two polymerases. The temperature sensitivity of Pol II condensates is particularly interesting, because this may position the Pol II foci as a temperature-responsive regulatory element, potentially enabling rapid transcriptional adaptation without global repression of rRNA production.

Our results extend our previous work showing that ATTF-6 is required for SL1 accumulation but is dispensable for 5S rRNA biogenesis (Wang et al. 2025). We now show that ATTF-6 is required for the assembly of Pol II foci at the 5S rDNA–SL1 locus, providing a mechanistic explanation for its selective effect on SL1 transcription. Pol III foci, by contrast, remain intact without ATTF-6, consistent with the preserved 5S rRNA levels upon ATTF-6 depletion (Figures 6 and 7). Although our analyses show a clear correlation between Pol II foci disassembly and reduced SL1 transcript levels, caveats remain. For example, SL1 and 5S RNA populations may differ in stability. Hence, unchanged 5S abundance under heat stress does not necessarily reflect maintained transcription. Future approaches that directly measure nascent RNA synthesis, such as metabolic labeling, will be important for resolving the transcriptional responses of both loci.

Together, our findings support a model in which ATTF-6–dependent Pol II foci drive efficient SL1 transcription, while Pol III independently forms its own foci to produce 5S rRNA (Figure 7G). The two polymerases coexist within a shared genomic compartment yet operate through distinct assembly principles and heat sensitivities. This organizational strategy may allow animals to tightly couple Pol II–driven SL1 production to physiological conditions while preserving robust Pol III–mediated 5S rRNA synthesis.

## Materials and Methods

### Maintenance of *C. elegans* strains

All strains were grown at 20°C unless otherwise stated. Wild-type refers to the N2 Bristol strain (Brenner 1974). GFP::AMA-1 and GFP::RPC-1 strains were obtained from the CGC and are referred to as GFP::POL II and GFP::POL III, respectively, for clarity throughout this manuscript. A complete strain list may be found in Supplementary Table S1.

### CRISPR/Cas9 genome editing

CRISPR/Cas9 genome editing in *C. elegans* was carried out as previously described (Kim et al. 2014). The ATTF-6::RFP and ATTF-6::AID::RFP strains were generated using AID::RFP double-stranded DNA donors in a *sun-1p*::TIR1 background. The RFP::Pol II and RFP::Pol III strains were produced using RFP double-stranded DNA donors in the GFP::ATTF-6 background. Donor DNA was mixed with a pre-assembled Cas9 ribonucleoprotein complex consisting of Cas9 protein, gRNA, and tracrRNA (IDT). The pRF4 plasmid carrying the dominant rol-6 allele was included as a co-injection marker (Kim et al. 2014). F1 roller progeny were selected and screened by PCR. Homozygous insertion strains were confirmed by Sanger sequencing. DNA donor templates, gRNA sequences, and genotyping primers are listed in Supplementary Table S2.

### DNA-FISH and immunofluorescence

The approach was adapted from an established protocol with modifications (Phillips et al. 2009; Adilardi and Dernburg 2022). Briefly, ~5000 gravid adult worms (ATTF-6::3×FLAG) were bleached and washed with Egg Buffer (EB: 25 mM HEPES-NaOH, pH 7.4, 118 mM NaCl, 48 mM KCl, 2 mM EDTA, 0.5 mM EGTA). Approximately 50 eggs in 20 μL EB were seeded onto a coverslip and fixed by adding 20 μL of 4% formaldehyde in EB to reach a final concentration of 2%, followed by incubation for 2–3 min. After fixation, the eggs were covered with a poly-L-lysine–coated slide. The slide was placed on a metal block precooled with dry ice for 30 min. The coverslip was then carefully flicked off, and the slide was immediately immersed in −20°C methanol for 10 min. Residual liquid was removed from around the eggs, and the samples were washed once with 2× SSCT (0.3 M NaCl, 0.03 M sodium citrate, pH 7.0, 0.1% Tween-20). The eggs were then incubated in 2× SSCT containing 50% formamide at 37°C overnight. For DNA probe hybridization, synthesized Cy5-labeled DNA probes (10 ng/μL; IDT) were diluted in hybridization buffer (HB: 3× SSC, 48% formamide, 10.6% dextran sulfate). The probe mixture was added to the samples, covered with a coverslip, and heated at 80°C for 10 min. Slides were then transferred to a light-protected humid chamber and incubated at 37°C for 6 h. After hybridization, egg samples were washed three times with PBST (PBS + 0.1% Tween-20) and incubated in blocking buffer (PBST containing 0.5% BSA) at room temperature for 1 h. Mouse anti-FLAG primary antibodies (Sigma-Aldrich) were diluted 1:500 in blocking buffer and applied to the samples overnight at 4°C. After three PBST washes, samples were incubated with goat anti-mouse Alexa Fluor 488 secondary antibody (1:500 in blocking buffer) for 2 h at room temperature in the dark. Following incubation, samples were washed three times with PBST. A drop (10 μL) of DAPI-containing mounting medium (Vector Laboratories) was applied to the eggs and incubated for 10 min at room temperature. Samples were then covered with a glass coverslip and sealed with nail polish.

### RNA interference by feeding of double stranded RNA

HT115 RNAi feeding bacteria were streaked from the Ahringer RNAi library as described previously (Kamath et al. 2003; Price et al. 2021). In brief, Nematode Growth Medium (NGM) plates containing 50 μg/mL ampicillin and 5 mM IPTG were seeded with HT115 bacteria expressing double-stranded RNA (dsRNA) targeting the gene of interest. L1 larvae were transferred onto RNAi plates and grown at 25°C for two days. Gravid worms were then dissected, and their embryos were imaged. L4440 RNAi was used as the control for all RNAi experiments.

### Auxin treatment

The auxin treatment was performed as previously described (Zhang et al. 2015; Wang et al. 2025). Briefly, gfp::tev::aid::attf-6; sun-1p::TIR1 reporter strains were plated on NGM plates containing 1 mM natural auxin indole-3-acetic acid (IAA) at the L3–L4 stage and incubated overnight at 25°C. Germlines of gravid adult worms were imaged to confirm complete protein depletion. The worms were then dissected, and their embryos were imaged.

### Granule disruption with hexanediol or heat stress

For 1,6-hexanediol (Sigma-Aldrich) treatment, L4 worms expressing GFP::ATTF-6, GFP::Pol II, or GFP::Pol III, respectively, were plated on *ptr-2* RNAi plates to permeabilize the eggshell of embryos for 1,6-hexanediol treatment and fed for approximately 24 hours (Thomas et al. 2023) At adulthood, *ptr-2* RNAi treated adults were dissected into either egg buffer (EB) or 5% 1,6-hexanediol in EB on ring slides and imaged.

To disrupt granules via heating, GFP::ATTF-6, GFP::Pol II, or GFP::Pol III expressing gravid adults grown at 20°C were bleached to collect embryos. For the control condition (20°C), resuspended embryos were mounted on glass slides with 4% agarose pads and imaged immediately. For heating, embryos were resuspended in ~ 4 mL M9 in a 15 mL conical tube and transferred to a 32°C incubator (VWR Scientific Model 1545) shaking on a nutating mixer (Fisher Scientific). Embryos were imaged at ambient temperature either 2 hours or 3 hours post-transfer to 32 °C.

### Northern Blotting

To assay overall ribosomal RNA abundance, 7.5 μg total RNAs were separated on a 8% polyacrylamide/7M urea gel and stained with ethidium bromide (Sigma).

To quantify SL1 or 5s rRNA abundance, 15 μg total RNAs were separated on a 8% polyacrylamide/7M urea gel and transferred to Hybond N+ membrane (GE healthcare) at 400 mA for 1 hour with 1x Tris-Borate-EDTA buffer. RNAs were crosslinked to the membrane using ultraviolet light (254 nm; 120 mJ) with a Stratalinker (Stratagene). Membranes were prehybridized with Ultrahyb Ultrasensitive Hybridization Buffer (Invitrogen). Subsequently, 10 μmol of either fluorescein amidite (FAM)-labeled anti-SL1 or anti-5s rRNA probes were hybridized to their respective membranes at 37 °C, rotating overnight. The following day, the membranes were washed three times with wash buffer (0.1% sodium dodecyl sulfate, 0.1% saline-sodium citrate) at 37 °C and visualized using a Sapphire Biomolecular Imager (Azure Biosystems). SL1 and 5S rRNA abundance was quantified using FIJI.

### Heat-stress conditions and RNA extraction

Samples were prepared for RNA extraction as follows. Wild-type gravid adults grown at 20°C were bleached to obtain embryos. For each condition, 600,000 embryos were resuspended with 9 mL M9 in 15 mL conical tubes. For the control condition (20°C), RNA was extracted from resuspended embryos following bleaching. For the heat-stress conditions, RNA was extracted from resuspended embryos transferred to 32°C (VWR Scientific Model 1545) and incubated on a nutating mixer (Fisher Scientific) for either 2 hours (32°C, 2 hours) or 3 hours (32°C, 3 hours). For all three conditions, respectively, samples were pelleted and total RNAs extracted using TriReagent (Sigma-Aldrich).

### RT-qPCR (Real-time quantitative PCR)

100 ng of total RNAs from embryos were reverse transcribed via Multiscribe Reverse Transcriptase (Thermo Fisher Scientific) using gene-specific antisense RT primers containing a universal stem loop. Real-time quantitative PCR was conducted with diluted RT reactions (1/4 for SL1, *act-3*, and *gpd-2*; 1/100 for 5S rRNA and 18S rRNA) using the respective gene-specific forward primer and an antisense primer matching the universal stem loop (Pastore et al. 2021). Reactions were conducted in triplicate using PowerUP SYBR Green Master Mix (Thermo Fisher Scientific) and run with the CFX Connect Real-Time PCR System (Bio-Rad). Transcript levels were normalized to the reference gene 18S rRNA, and relative levels upon heating was calculated via the ΔΔCt method. Primer sequences listed in Supplementary Table S2.

### Analysis of ChIP-seq data

TrimGalore was used to trim adapter sequences and filter out low-quality reads(Kechin et al. 2017). The resulting high-quality reads were aligned to the genome with the BWA-MEM algorithm under default settings (Vasimuddin et al. 2019). Following alignment, BAM files were transformed into BigWig files using DeepTools bamCoverage function(Ramírez et al. 2016). Enrichment peaks from ChIP-seq data were detected with MACS2 employing the narrow-peak option (Zhang et al. 2008; Feng et al. 2012). BigWig signals from two biological replicates were averaged using the DeepTools bigwigMerge function. To compare IP and control signals, experimental BigWig files were subtracted from control BigWig files using bigwigCompare with the parameters (-p max -operation subtract - pseudocount 1 -binSize 5). Further processing and statistical analyses were carried out with custom R and Python pipelines. Visualization of BigWig files was performed using IGV (Robinson et al. 2011). ChIP-seq metagene profiles were generated using the SeqPlots software (Stempor and Ahringer 2016). The subtracted BigWig files from the ChIP-seq pipeline served as input, and BED files containing SL1 snRNA genes were used as references for the plots.

### Microscopy

Unless otherwise indicated in the figure caption, all images depict live embryos dissected from gravid adults into EB and mounted on glass slides with 4% agarose pads.

Most imaging was conducted using the Nikon Ti2 inverted microscope equipped with an X-Light V3 spinning disk confocal unit (CrestOptics) in NIS-Elements AR 5.41.02, using the Plan Apo 60x water objective (Price et al. 2023) with the following exception:

To determine fluorescence recovery after photobleaching (FRAP) of ATTF-6, Pol II, and Pol III foci in embryos, spinning disk confocal images were taken using a Nikon TiE inverted microscope equipped with an Andor Revolution WD spinning disk system. Images were captured with a CFI Plan Apo VC 100x/1.4 NA oil immersion objective and Andor iXon Ultra897 camera. All further image processing was conducted with FIJI.

### Image Quantification

To quantify Roundness, 3 embryos at the ~20-30 cell stage were selected per strain. Spinning disk maximum intensity projections (60x) spanning the top layer of embryonic nuclei (9.6 μm; step = 0.6 μm) were used for quantification. First, background fluorescence was removed using a rolling-ball subtraction radius of 140 μm. Foci were selected via the following thresholds: 25-100% of maximum pixel intensity for ATTF-6 and Pol III; 65-100% for Pol II. A more stringent threshold was used for Pol II to isolate the brighter, larger foci at the splicing-leader cluster and to account for the higher background cytoplasmic intensity of GFP::AMA-1. To exclude noises, only particles 0.05-0.6 μm^2^ were analyzed. Roundness was quantified with the Analyze Particles tool in FIJI. Roundness=4x Areaπx(major axis)2. The diameters of ATTF-6, Pol II, and Pol III foci were measured in FIJI by manually drawing a line across the diameter of each focus and measuring the distance, using the same images as those used for roundness quantification.

Photobleaching of GFP::ATTF-6, GFP::Pol II, or GFP::Pol III foci was conducted using live embryos dissected into EB. Foci were photobleached using the FRAPPA module in MetaMorph with a 70% 488 nm laser, and a dwell time <1 ms per pixel, and either a single pulse (ATTF-6) or two pulses (Pol II and Pol III) using a rectangular ROI encompassing the foci. Images were acquired every second for 5 seconds prior to and 30 seconds post photobleaching. Images were further analyzed in FIJI.

For each focus, the fluorescence intensity at each timepoint (It) was background-corrected with intensity outside the embryo (I_b_). The mean pre-bleach intensity (I_0_) was calculated from 5 frames prior to bleaching, and corrected to I_b_. The normalized fluorescent intensity was then computed as: ${\text{I}}_{\text{norm}}\left(\text{t}\right)\frac{\left(I_{t}-I_{b}\right)}{\left(I_{o}-I_{b}\right)}$.

To determine whether ATTF-6, Pol II, or Pol III exhibited measurable recovery, the maximum post-bleach intensity was compared to the baseline intensity at time zero. Recovery was considered significant if the increase in intensity exceeded twice the standard deviation of the baseline, to avoid fluctuations due to noise. For proteins that met the above criteria, the mean I_norm_ was calculated at each timepoint, and the mean value at the first post-bleach frame was defined as y_0_. FRAP recovery was modeled in R with a first-order exponential function: $\text{F}\left(\text{t}\right)={\text{y}}_{0}+{\text{A}}_{\mathrm{rec}}*\left(1-{\text{e}}^{-k\text{Time}}\right)$, where A_rec_ is the fluorescence recovery amplitude and ***k*** is the recovery rate constant. Curve fitting was performed using the Levenberg–Marquardt algorithm implemented in the nlsLM function from the minpack.lm R package. t_1/2_ was calculated from the first order exponential model.

To quantify the number of foci per nucleus for GFP::ATTF-6, GFP::Pol II, or GFP::Pol III embryos, maximum intensity projections spanning the top single layer of nuclei (6.6 μm, step=0.3 μm) were generated. Circular ROIs were drawn surrounding each nucleus and foci were manually counted using the Multipoint tool in FIJI. The number of points bound by each ROI was calculated in FIJI to calculate foci numbers. Three embryos were quantified per condition.

## Supplementary Material

1

## Data Availability

ATTF-6 ChIP-seq data are available at NCBI under the accession number GSE277641 (Wang et al. 2025). Pol II and Pol III ChIP-seq data are available at NCBI under the accession number GSE42741 (Ikegami and Lieb 2013).
