# Supplementary material for: Dynamic RNA Polymerase compartments organize the transcription of gene clusters
Source: bioRxiv. 2026 Jan 8:2026.01.07.698080. Preprint. [Version 1] doi: 10.64898/2026.01.07.698080 (PMC12803116; doi:10.64898/2026.01.07.698080)
Supplement: 1 [file NIHPP2026.01.07.698080v1-supplement-1.pdf]

bioRxiv preprint doi: https://doi.org/10.64898/2026.01.07.698080; this version posted January 8, 2026. The copyright holder for this preprint (which was not certified by peer review) is the author/funder, who has granted bioRxiv a license to display the preprint in perpetuity. It is made available under aCC-BY 4.0 International license.

Figure S1. RNA Pol II and Pol III foci are formed at the 5S rDNA–SL1 locus in the germline.

(A) Schematic of the *C. elegans* germline and embryos in utero. Colored lines highlight the germline at different developmental stages. Homologous chromosomes undergo synapsis during early-mid pachytene.

(B) Fluorescent images of germ nuclei in worms expressing ATTF-6::RFP and GFP::Pol II at early-mid pachytene and late pachytene. Each dotted circle marks a germline nucleus. Confocal images (60× objective) are shown as maximum-intensity projections spanning the germline. Arrows indicate the piRNA gene cluster, and arrowheads mark the 5S rDNA-SL1 gene cluster. Scale bar: 2 μm.

(C) Same as (**B**), but with GFP::Pol III.

(D) Fluorescent images of germ nuclei in worms expressing mCherry::PRDE-1 and GFP::Pol II at early-mid pachytene and late pachytene. Each dotted circle marks a germline nucleus. Confocal images (60× objective) are shown as maximum-intensity

bioRxiv preprint doi: https://doi.org/10.64898/2026.01.07.698080; this version posted January 8, 2026. The copyright holder for this preprint (which was not certified by peer review) is the author/funder, who has granted bioRxiv a license to display the preprint in perpetuity. It is made available under aCC-BY 4.0 International license.

projections spanning the germline. Arrows indicate the piRNA gene cluster, and arrowheads mark the small GFP::Pol II foci. Scale bar: 2 μm.

(E) Same as (**D**), but with GFP::Pol III.

Figure S2. ChIP signals of ATTF-6, RNA Pol II, and RNA Pol III across genome.

(A) Browser view of ATTF-6, Pol II (AMA-1), and Pol III (RPC-1) ChIP-seq signals on chromosome I. ATTF-6 ChIP-seq signals were normalized to the control IP, and AMA-1 and RPC-1 signals were normalized to the input by subtracting their corresponding control signals. The signals represent the average from two biological replicates and are shown in RPKM (Reads Per Kilobase per Million mapped reads).

(B–E) Browser views for chromosomes II, III, IV, and X.

Figure S3. Quantification of ATTF6, Pol II, and pol III foci number in Auxin or RNAi treatment.

(A) Definition of foci patterns used for quantifying the foci in (**B–E**), including two clear foci, multiple small foci, and dissolved foci.

(B) Quantification of GFP::Pol II and ATTF-6::RFP foci patterns in embryo nuclei based on the definitions in (**A**). Embryos were dissected from worms treated with or without auxin (1mM IAA). Nuclei from three independent embryos were counted, and the number of nuclei is indicated (n = x).

(C) Same analysis as in (**B**), but examining GFP::Pol III and ATTF-6::RFP foci.

(D) Quantification of GFP::Pol II and ATTF-6::RFP foci patterns in embryo nuclei based on the definitions in (**A**). Embryos were dissected from worms treated with control (L4440), *attf-6*, and *snpc-4* RNAi. Nuclei from three independent embryos were counted, and the number of nuclei is indicated (n = x).

(E) Same analysis as in (**D**), but examining GFP::Pol III and ATTF-6::RFP foci.

Figure S4. Number of ATTF6, Pol II, and pol III foci per nucleus.

(A-C) Violin plots showing the number of foci per nucleus for embryos expressing GFP::ATTF-6 **(A)**, GFP::Pol II **(B)**, or GFP::Pol III **(C)**. Embryos resuspended in M9 buffer were maintained at 20°C or exposed to heat-stress at 32°C. Time (hrs) indicates time

bioRxiv preprint doi: https://doi.org/10.64898/2026.01.07.698080; this version posted January 8, 2026. The copyright holder for this preprint (which was not certified by peer review) is the author/funder, who has granted bioRxiv a license to display the preprint in perpetuity. It is made available under aCC-BY 4.0 International license.

post-transfer to a 32°C incubator. Dots represent the median foci number and n represents individual nuclei. Three embryos were quantified per condition. Statistics conducted using the Student’s t-test (ns, not significant; **** $p \leq 0.0001$).
